# Supplementary material for: Are carbon emissions trading and green financial instruments synergistic? -Comprehensive quantitative research based on content analysis
Source: PLoS One. 2024 Mar 7;19(3):e0298601. doi: 10.1371/journal.pone.0298601 (PMC10919601; doi:10.1371/journal.pone.0298601)
Supplement: S1 Data — (ZIP) [file pone.0298601.s001.zip › Data/地级市Z_F工作报告.zip!/╔╜╢1⁄2/╤╠╠¿╩╨/└┤╫╘╣1⁄2╓┌║┼í╛┬φ┐╦╩2╛▌═°í┐_2005.docx]

各位代表：

现在，我代表市政府，向大会作政府工作报告，请予审议，并请政协各位委员和其他列席人员提出意见。

一、2004年政府工作回顾

2004年是不同寻常的一年。一年来，全市人民群众在高举邓小平理论和"三个

代表"重要思想伟大旗帜，以党的十六大和十六届三中、四中全会精神为指导，在中共烟台市委的正确领导下，牢固树立和认真落实科学观，全面贯彻国家宏观调控政策和省委、省政府各项决策部署，解放思想，迎接挑战，干事创业，加快发展中得到更多实惠。，较好地完成了十四届人大二次会议确定的各项任务。

———积极推进跨越发展，经济呈现既快又好的发展态势。2004年，全市城市居民人均可支配收入达到10803生产总值初步核算为1639亿比上年增长18.8%，比全省平均增幅高3.8个百分点工业完成增加值839.7亿元，农民人均纯27.8%，规模以上工业实现利税285.6亿元、利润199.5亿60.4%和68.8%。地方财政收入完成64亿元，增长33.8%。全社会固定资产投资完成1085亿元，增长47.2%。总投资千万元以上项目完成投资584.4亿元，增长33.1%。

———大力调整经济结构，优势产业和骨干企业进一步凸现。继续推进农业产业化经营，不断优化农业内部结构，水果、水产、畜牧、蔬菜、食用菌等高效产业规模进一步扩大，实现产值占农业的比重达到80%。大力推进半岛制造业基地暨北部沿海经济产业带建设，突出发展四大支柱产业和十大产品集群，工业结构升级，产业特色形成。全市规模以上工业完成销售收入2518亿元，增长43.6%；四大支柱产业完成销售收入1329亿元，占规模以上工业的比重达到52.8%；全市销售收入过10亿元的企业由20家增加到30家，利税过亿元的企业由24家增加到48家。高新技术产业产值达到629.9亿元，占规模以上工业的比重由20.7%提高到23.7%。东岳汽车、浪潮LG、玲珑橡胶等项目迅速膨胀，南山铝板带箔、16万吨MDI、鸿富泰电子等新建项目以及海阳核电、海水淡化等前期项目顺利推进。积极培育大型流通骨干企业，加快发展旅游业、物流业，启动夜间经济，服务业保持了良好的发展势头。国内外著名的百盛、东方家园、国美、澳柯玛等商业企业入驻我市。全市服务业增加值完成543.1亿元，增长14.5%；社会消费品零售总额达到440.4亿元，增长16.7%；旅游业总收入达到93亿元，增长30%。金融保险、交通运输、邮政通信、中介咨询、社区服务等行业健康发展。

———着力提升开放层次和水平，对外经贸取得新的突破。全市合同利用外资42亿美元，实际利用外资21.4亿美466084%和92.1%。外贸进出口总额完成79.9亿美增长35.7%，其中出口45亿美元，35%。机电产品、高新技术产品出口增幅分别达到60.2增长10.4%和13.0.3%，创历史最好水平。加工贸易增势强劲，占全市出口总额的比重由上年的44.4%提高到50%。"走出去"步伐明显加快，对外经济技术合作取得新的进展。各类园区在规范整顿中得到较快发展，经济技术开发区经济总量在全国49个国家级开发区中由第8位前移至第7位，出口加工区B区基础设施建设基本完成。12个省级以上园区实际利用外资和外贸出口分别占全市总额的34.8%和51%。国内合作成效显著，全市共引进内资338亿元，增长16全年新增城镇就业再就业10.8万人对外交往日益活跃，与友好城市的合作取得实效。APEC电子商务博览会、第六届国际果蔬博览会等重大经贸活动取得圆满成功，城市知名度和影响力明显提高。

———继续深化市场取向的各项改革，经济发展活力明显增强。积极稳妥地推进国有企业改革。市属大企业改革迈出较大步伐，实现了国有资产的有序进退、保值增值。困难企业改革力度加大，较好地维护了职工权益。大力开拓资本经营，绿叶制药、山东吉龙两家公司股票在新加坡成功上市，我市上市股票总数达到12支，全年募集资金11亿元。民营经济快速增长，占GDP的比重达到69镇登记失业率控制在3.4%。农村税费改革深入推进，取消了农业特产税并下调农业税率3个百分点，减轻农民负担3.2亿元。国家对种粮农民的补贴政策全部落实到位，当年落实补贴资金2582万元。粮食购销市场化和国有粮食企业改革取得突破。财税体制改革成效明显，分配体制和支出结构更加优化。出口退税改革顺利实施。国土资源管理体制改革基本到位，规范整顿土地市场效果显著。市县机构改革、行政审批制度、投资体制、市政公用事业、文化卫生体制改革稳步推进。

———进一步加大城市建设力度，基本实现了"三年见成效"的目标。按照打造现代化城市的标准，努力提升城市内涵，改善城市环境，城市整体形象有了较大改观，荣膺"最佳中国魅力城市"，获得"中国人居环境奖"。全年完成城乡建设投入60亿元，其中中心城市完成20亿元。滨海广场、南大街、滨海东路等新建和改造工程基本完成，只楚路综合整治、二马路拓宽改造工程完工，国道206线烟蓬段改造、养马岛大桥建成通车，为城市增添了新亮点。渤海天然气开始向工业企业供气并具备向市区供气条件，烟大铁路轮渡工程全面开工，烟台港西港区建设前期工作积极推进。老城区改造力度进一步加大，部分社区街道、公共场所得到有效整治，市民的居住和生活环境明显改善。完善了城镇空间、生态绿地和产业布局以及路网结构规划，科学合理的规划体系初步形成。农村城市化步伐加快，县级市和小城镇建设成效明显，全市城市化水平达到51%，比上年提高2个百分点。

———坚持以人为本的执政理念，人民群众在发展中得到更多实惠。全市城市居民人均可支配收入达到10803元，农民人均纯收入达到4660元，分别增长10.4%和13.1%。全年新增城镇就业再就业10.8万人，城镇登记失业率控制在3.4%。全面推行了工伤和女工生育保险制度，“五位一体”的社会保障制度全面建立，城市居民最低生活保障标准由220元提高到260元。爱心捐助活动深入开展，新型农村合作医疗试点继续深化，拖欠职工和农民工工资问题得到较好解决，探索建立了失地农民基本生活保障制度。解决了87个村、5.9万人的饮水困难，完成了历史性缺水村人畜饮水解困任务；新增乡村道路硬化里程1375公里，新增通车村60个；投入农网改造资金5.3亿元，农网供电能力增加10%。尽心竭力为群众办好事、解难题，年初确定的为民服务20项实事圆满完成。

———大力实施可持续发展战略，各项社会事业协调推进。全年新增高新技术企业40家，有142项科技项目列入国家和省科技计划，荣获“全国科技进步先进城市”称号。基础教育质量稳步提高，高中阶段教育得到加强，高等教育办学规模进一步扩大，职业教育改革纵深推进。外商、外来人口和农村进城务工人员子女就学环境明显改善。文化创作成果丰硕，群众性文化活动广泛开展。疾病预防控制体系和医疗救助体系不断完善，公共卫生信息网络系统建设初步完成。体育工作再创佳绩，我市运动健儿在第28届奥运会上获得2枚金牌、1枚银牌，打破5项世界纪录。新一轮创建全国双拥模范城活动深入开展，双拥共建工作再上新水平。着力建设“生态城市、绿色烟台”，环境保护工作取得新成效。全市新增造林面积11.3千公顷，市区新增绿地40公顷。全面落实各项奖励政策和技术服务措施，人口与计划生育工作水平进一步提高。老龄、妇女、儿童和残疾人事业得到加强，广播电视、新闻出版、气象地震、史志档案、外事侨务、民族宗教等工作取得新的成绩。

———不断加强精神文明和民主法制建设，促进了社会文明进步和安定团结。以争创全省全国文明城市为总目标，深入开展“我为烟台添魅力”以及建设文明社区、文明村镇等群众性精神文明创建活动，进一步加强“诚信烟台”建设，城乡文明程度明显提高。围绕创建全国最安全城市，不断加强社会治安综合治理，着力构建矛盾纠纷疏导控制体系，大力整顿规范市场经济秩序，强化安全生产监督管理，全年没有发生重大安全生产事故，保持了政治稳定、社会安宁。坚持依法行政，主动接受人大、政协及各民主党派、工商联和社会各界的法律监督、工作监督和民主监督，高度重视人大代表建议和政协提案的办理工作。认真贯彻实施《行政许可法》，积极推进“阳光政务”工程，决策公示制、预告制、通报制进一步完善，办事效率和服务质量明显提高。认真落实领导干部廉洁自律各项规定，严肃查处违法违纪案件，严格纠正损害群众利益的不正之风，树立了政府机关和公务员队伍的良好形象。

回顾一年来的工作，我们深切体会到，面对各种机遇和挑战，面对跨越发展的更高要求，要不断开创改革开放和现代化建设的新局面，必须坚持用科学发展观统揽全局，用统筹的观念、科学的方法、实干的精神，推动经济社会全面、协调、可持续发展；必须在解放思想中统一思想，把国家宏观调控政策与本地实际结合起来，创新思路，科学运筹，以调控促改革、促开放、促调整、促发展；必须善于把握全局，突出关键环节，在事关全局和长远发展的重大战略和重大项目上，实施重点突破；必须坚持和实践以人为本的执政理念，真心实意关心人民生活，千方百计解决群众困难，使人民群众在发展中得到更多实惠；必须在党的领导下，坚持科学行政、民主行政、依法行政，强化社会管理和公共服务职能，不断提高政府工作的能力和水平；必须大力营造加快发展的浓厚氛围，保护好、引导好、发挥好各方面的积极性，不断激发你追我赶、干事创业的发展热情。这些都是全市上下在实践中探索和积累的经验体会，我们要在今后工作中大力发扬，不断丰富完善。

各位代表，过去一年的成绩来之不易。这是全市人民在省委、省政府和市委的正确领导下，开拓奋进、共同努力的结果，是社会各界和海内外友好人士热情参与、积极支持的结果。在此，我代表市政府，向全市广大工人、农民、知识分子以及各阶层的建设者，向各位人大代表和政协委员，向各民主党派、各人民团体、社会各界人士，向驻烟人民解放军、武警部队官兵和公安干警，向中央、省属驻烟单位，向所有关心支持烟台建设的港澳同胞、台湾同胞、海外侨胞和国际友人，致以崇高的敬意和衷心的感谢！

2004年我市经济社会发展取得了令人鼓舞的成绩，但也存在一些突出问题：经济发展的结构性、体制性、机制性矛盾依然存在，骨干企业膨胀不快，辐射带动作用不强，服务业发展相对滞后；社会保障体系还不够完善，农民土地征用、城市居民拆迁、企业改制破产等工作中仍存在不少矛盾，化解机制还有待完善，部分群众生产生活中的困难和问题解决得还不够好；各级政府及其部门职能和作风转变还没有到位，部分工作人员全局观念淡薄、服务意识不强、法律素质不高，形式主义、官僚主义以及行政不公、为政不廉等现象不同程度地存在。我们一定要正视这些问题，下决心认真加以解决。

二、2005年政府工作安排

2005年是“十五”计划的最后一年，也是保持经济社会良好发展势头、推动跨越发展的关键一年。做好今年的工作，对全面实现“十五”计划目标，为“十一五”期间经济社会发展奠定坚实基础，具有十分重要的意义。当前，世界经济持续增长，国内经济活力明显增强，我市加快发展的思想基础进一步巩固，经济的内在增长机制明显向好，经济社会发展进入了市场推动和政府调控良性互动的新阶段。但与此同时，一些制约发展的矛盾和亟待解决的问题还比较突出，区域之间、城市之间的竞争将更加激烈。我们必须不断增强机遇意识、忧患意识和创新意识，解放思想，开拓进取，因势利导，发奋工作，努力开创全市经济社会发展的新局面。

今年的政府工作，要以邓小平理论和“三个代表”重要思想为指导，全面贯彻党的十六大和十六届三中、四中全会精神，紧紧抓住并切实用好重要战略机遇期，以科学发展观统领全局，认真落实省委、省政府“东部突破烟台”的决策部署，紧紧围绕“三个明显、两个高于、一个跨越”的目标要求，大力实施赶超战略，更加注重解放思想，更加注重结构调整，更加注重改革开放，更加注重可持续发展，正确处理改革发展稳定的关系，着力提升区域经济的整体竞争力，着力解决经济社会发展中的主要矛盾，着力解决关系人民群众切身利益的突出问题，努力实现全市经济持续快速协调健康发展和社会全面进步。

经济和社会发展的主要预期目标是：全市生产总值增长14%；地方财政收入增长20%；全社会固定资产投资增长22%；社会消费品零售总额增长15%；城镇居民人均可支配收入增长8%，农民人均纯收入增长6%；城镇登记失业率控制在3.5%以内。

按照上述目标，今年要集中抓好以下工作重点：

（一）进一步推动产业结构优化升级，构建更具竞争力的现代产业体系

紧紧围绕胶东半岛制造业基地和北部沿海经济产业带建设，集中力量培育“三个一批”，提升工业规模和层次。一是培育一批支柱产业。突出四大支柱产业和十大产品集群，搞好产业配套，拉长产业链条，凸现产业优势，增强集聚效应。重视发展装备制造业，提升我市制造业科技装备水平。依托港口优势，大力发展临港工业，积极推进以港兴市。二是培植一批骨干企业。实施大企业大集团战略，引导各类生产要素聚集，着力引进具有战略性、全局性带动作用的大项目，推动优势企业快速扩张，逐步形成一批规模大、实力强、在行业中居于领先地位的骨干企业。力争汽车、手机、电脑三大集群率先实现突破，全面完成“2358”工程目标任务。三是培植一批知名品牌。积极引导和支持优势骨干企业加强品牌宣传，搞好品牌培育，在做大生产规模的同时做强品牌，力争获得更多的中国名牌或驰名商标。要大力支持有条件的企业开展品牌经营，放大品牌效应，争创国际品牌。优先发展高新技术产业，加快用高新技术改造传统产业步伐，努力争取在新材料、电子信息、生物技术及制药、机电一体化等四大领域，形成具有特色的产业集群。年内，全市规模以上工业增加值增长25%以上，高新技术产业占工业的比重达到25%以上。

突出发展服务业，推动服务业规模扩大、档次提升。一是突出发展商贸物流业。组织实施好市区商业网点发展规划，深入推进“三个十”工程，培植大型流通企业、骨干市场，繁荣夜间经济，拓展消费领域，培育新的消费热点。大力发展连锁经营、物流配送、电子商务等新型流通业态，高标准建设特色专业物流园区，争取进入国家首批保税物流中心（B型）试点城市行列。二是突出发展旅游业。精心打造滨海生态旅游胜地和“山海风情、人间仙境”的城市品牌，抓好旅游资源和休闲度假设施的开发整合，丰富旅游产品，优化要素结构，充分发挥各旅行社和旅游组织的作用，拓展海内外客源市场，做大做强旅游产业。年内，旅游业总收入达到115亿元，增长23%以上。三是突出发展交通运输业。完善以高等级公路为主框架的公路网络，调整优化全市港口布局，积极开辟海上、空中运输航线，努力构建现代交通运输体系，提高城市综合运输能力。同时，积极发展金融、中介、信息咨询和社区服务等现代服务业。年内，全市服务业增加值达到630亿元，增长16%。

进一步优化投资结构，加强经济社会发展中的薄弱环节和关键环节。集中力量抓好365个投资过亿元的投资项目，加快推进烟大铁路轮渡、国道206线（蓬莱至龙口段）改造工程、龙烟铁路、海水淡化、胶东引黄调水、烟台港西港区等重大基础设施项目，集中力量抓好海阳核电、国电蓬莱发电厂、东岳汽车扩建、鸿富泰工业园、环日子午轮胎等重大产业项目。大力拓宽融资渠道，引导外资、民资扩大投入，积极争取国债项目，鼓励金融机构在继续加大对重点产业、企业、项目支持的同时，增加对中小企业和民营企业的信贷投放，推进优质企业境内外上市和上市公司再融资。规范政府性投资项目决策和实施程序，对非政府投资的项目，不再实行审批制，根据国家有关政策实行核准制或备案制。进一步加强土地管理，做到规范化征用土地、集约化利用土地、市场化经营土地，提高土地利用效益。

（二）进一步加大对“三农”的支持力度，保持农业和农村发展的良好势头

推进农业结构战略性调整，大力发展现代农业。推广优质专用粮油品种，膨胀发展果品、水产和畜牧业。继续深入开展“中国食品名城”创建活动。抓好国家确定的苹果、水产品优势产区建设。大力扶持农业产业化龙头企业，积极培育农村专业合作经济组织和行业协会，搞好农产品的精深加工和市场流通。健全农产品质量标准体系和检测体系，完善动物疫病防治体系，积极发展无公害农产品、绿色食品和有机食品，提高农产品的质量安全水平，增强国际竞争力。年内，全市农副产品出口16.5亿美元，增长20%。

加快农村城市化进程，提高农村文明程度。进一步加大投入，抓好农村基础设施、商贸设施、文化设施和保障体系建设。年内投资3.9亿元，改建硬化农村公路1000公里以上，完成行政村基本通油路工程；启动村村通自来水工程，力争用3年时间，彻底改善全市农村人口的饮水状况；启动村镇环境综合整治，今年先在中心镇、城市近郊村镇和高等级公路、铁路等主要交通干道两侧500米范围内的村镇展开，积累经验后再全面推广。遵循“依法、自愿、有偿”的原则，推进农村土地使用权的合理有序流转。大力发展农村二三产业，鼓励更多的企业和农村劳动力向园区流动。优化配置农村劳动力，强化农民科技教育培训，安排200万元用于农村劳动力转移培训的“阳光工程”，建立完善农村劳动力就业服务体系。

狠抓农村政策落实，继续加大对“三农”的支持力度。全面落实对种粮农民直补、良种补贴和购置大型农机具补贴等各项支农政策，年内全部取消农业税和农村义务工。启动“农业科技入户工程”，支持引导农业科研、技术推广部门与龙头企业、农产品基地合作。抓好农业综合开发，加强农机、水利等农业基础设施建设，实行严格的耕地保护制度，稳定和加强粮食生产。继续实施镇村“双十”、“双百”工程，带动农村经济社会全面协调发展。

（三）进一步扩大对外开放，不断提高经济国际化水平

适应入世“后过渡期”对外开放的新形势，不断提高利用外资质量和水平。以国家产业政策和我市产业发展规划为导向，突出高科技型、环保型、节能型先进制造业，突出金融、物流、旅游、文教等现代服务业和城市基础设施、公用设施，突出标准化、大规模种养业和农产品加工业，不断拓展招商领域。在主攻日韩、港台的同时，扩大利用欧美资本规模；在抓好直接利用外资的同时，积极采用跨国并购、境外上市、BOT、政府贷款、国际金融组织融资等途径，不断拓展引资市场和渠道。完善以政府为主导、企业为主体的招商引资机制，积极推行产业招商、专业招商和代理招商。充分发挥各类园区的示范和载体作用，加强政策引导和管理，创新机制，完善功能，突出特色，不断提高发展水平。年内，全市实际利用外资25亿美元，增长25%。高度重视内资引进，以珠三角、长三角、京津和东北地区为重点，积极吸引国内大企业、大集团入驻我市。年内，全市实际利用内资达到420亿元，增长25%。着力打造产业环境，优化服务环境，改善法治环境，提升人文环境，不断提高环境吸引力和竞争力。

深入实施科技兴贸和以质取胜战略，加快转变外贸增长方式。在巩固农副产品、纺织服装出口的同时，扩大机电产品、成套设备和高新技术产品出口，大力发展加工贸易并促其转型升级。实施出口市场多元化战略，巩固日韩等传统市场，大力开拓欧美、非洲及南亚等新兴市场。改善进口商品结构，扩大先进技术、关键设备、重要物资和原材料进口。年内，全市外贸出口52.6亿美元，增长17%。

积极推动“双向”开放，大力实施“走出去”战略。完善境外投资扶持政策和服务体系，鼓励有竞争优势的行业以成熟技术和设备，向新兴市场转移，开拓新的发展空间；支持具备条件的企业到境外建立长期稳定的能源原材料基地，利用当地资源，满足发展需要。

（四）进一步深化各项改革，不断创新全面协调可持续发展的体制机制

深化国资国企改革。按照权利、义务和责任相统一，管资产和管人、管事相结合的原则，健全国有资产监管营运体系，完善国有出资人制度。坚持有进有退、合理流动的原则，调整优化国有经济布局，促进国有资本向先导性、基础性、公益性领域集中，推动重点骨干企业实施更大范围、更高层次的资产重组和资本运作，促其快速发展。加快产权制度改革，积极推进大企业的主辅分离、辅业改制和主业投资主体多元化。采取多种形式，搞好困难企业的改革解困。加强对已改制企业的后续管理和服务，促其完善法人治理结构，加快建立现代企业制度。年内重点抓好60户市属企业产权改革。

推进各项综合配套改革。以强化财政管理和公平预算分配为重点，全面深化财政体制改革，落实国库集中支付、政府采购和收支两条线制度；综合运用财政贴息、奖励等形式，扶持有利于增加财政收入的优势产业和项目，不断培植新的财政增长点。坚持依法治税管税，加强社会综合治税，进一步强化税收征管，管好非税收入，做大财政收入盘子。加快现代市场体系建设，积极发展资本、土地、技术和劳动力等市场，促进生产力要素合理流动。按照政事分开、事企分开的原则，加快推进事业单位改革，继续推进公交、供水、供热、供气等公用事业领域改革。

大力发展民营经济。积极营造浓厚的创业环境，形成崇尚创业、致富光荣的良好氛围。进一步放宽市场准入，保障民营经济与其他所有制经济享有同等的发展机会。扶持发展科技型、出口型、配套型企业和有较强拉动力的规模型企业。继续实施“私营企业进园区，个体工商户进市场”活动，推动产业集聚，壮大企业集群。搞好民营经济融资担保、信用评价、人才培训、技术信息、法律服务和行业协会等体系建设，为民营企业提供优质服务。全市民营经济实现增加值达到1370亿元，增长20%。

（五）进一步协调城乡发展，提升区域综合竞争能力

按照现代化城市的标准和要求，围绕树立“魅力城市”、“生态城市”、“开放城市”形象，坚持高起点规划、高标准建设、高效能管理，推动城市建设向“五年大变样”目标迈进。集中政府资金，引入外资和社会资金，重点抓好滨海景区、幸福、港站和文化广场等区片的改造建设，加快体育公园、学院路建设，搞好滨海路等重要路段的绿化美化和设施配套。加快推进世贸中心、韩国产业转移配套社区、市区文化休闲娱乐中心、大南山旅游风景区、雨岱山主题公园等经营性设施建设。继续实施老城区环境综合整治，不断改善居民生活和居住环境。进一步发挥规划的龙头作用，扩大控制性详规覆盖面，强化规划约束力。按照“管理重心下移”和“责权统一”的原则，继续深化城市管理体制改革，完善城市管理行政执法体系，强化城市街道社区管理服务功能，提高城市管理规范化水平。

紧紧抓住建设山东半岛城市群的战略机遇，推动中等城市崛起和县域经济发展。科学制定7个县级市城市发展规划，加大基础设施建设力度，强化园区建设和产业带动，迅速扩大城市规模，形成具有较强辐射带动能力的次区域性经济文化中心，努力构建布局合理、功能互补、特色鲜明的中等城市发展格局。建立健全跨行政区域的协调合作机制，突出强市，扶持弱市，突破中游市，搞好产业分工，有效整合资源，统筹发展县域经济，努力实现跨越赶超。

加快生态市建设，着力改善城乡生态环境。大力推进结构性污染治理，改造淘汰原材料和能源消耗高、污染严重的企业，严格点源治理，严格建设项目环境影响评价和“三同时”制度。加强重点流域、重点区域的污染综合治理，提高城市污水和垃圾集中处理率，提高人居环境质量。重视农业和农村污染防治，逐步改善农村环境面貌。加大造林绿化力度，搞好生态建设与保护。增强海洋国土意识，强化海洋综合管理，保护海洋生态环境。推行清洁生产，发展循环经济，努力建设资源节约型社会。

（六）进一步统筹各项社会事业，促进社会全面进步

加快发展教育事业，培养适应性建设人才。加强城乡统筹，调整基础教育布局结构，高标准、高质量、均衡化发展义务教育。支持各类高校扩大招生规模，提高办学水平。深化教育管理体制改革，鼓励发展民营教育。适应产业结构调整需要，大力发展职业教育。加快人才工作机制创新，着力培养企业经营管理人才、专业技术人才和高级技工人才。强化留学人员创业园区、博士后工作站、三校科技园等载体建设，完善技术、知识、管理等要素参与分配的制度，努力营造优秀人才脱颖而出、充分施展才能的良好环境。

积极推进科技进步，增强科技创新能力。加大科研投入和高新技术风险投资，围绕关键技术开展研发攻关，力争在重点领域取得突破，增强自主创新能力。积极搭建网络化数字设计与制造业信息化平台，健全技术研究和开发体系，培育、引进一批高层次工程技术研究中心，增强企业的核心技术优势。充分发挥驻烟高等院校、科研院所的作用，整合共享资源，深化产学研结合，加快科技成果转化。切实保护知识产权，维护知识产权权利人的合法权益。

繁荣各项社会事业，丰富群众文化生活。完善疾病预防控制体系、突发公共卫生事件医疗救治体系，积极发展社区卫生服务，稳步扩大新型农村合作医疗试点，全面加强农村卫生工作。进一步加强人口与计划生育工作，认真应对人口出生高峰期，人口自然增长率控制在2.4‰以内。积极备战第21届省运会，进一步提升我市竞技体育的整体实力。加快实施全民健身工程，兴建一批全民健身点。整合文化资源，发展文化产业。抓好艺术生产和文化交流，组织好重大文化活动。繁荣文学艺术、社会科学、新闻出版和广播电视事业，做好史志档案和文物保护工作，高度重视民族宗教、老龄和残疾人事业。

（七）进一步强化富民安民意识，不断提高人民生活水平

抓好就业再就业。贯彻实施《山东省就业促进条例》，落实就业目标责任制。注重发展个体私营经济和劳动密集型产业，不断扩大就业容量。认真落实各项再就业优惠政策，扩大再就业优惠政策享受范围，鼓励失业人员灵活就业、自主创业。做好困难群体再就业援助工作，确保有就业愿望的困难群体有就业岗位。建立健全就业服务体系，加大再就业培训和创业培训力度，缓解就业结构性矛盾。构建城乡统一的劳动力市场，加强对农村富余劳动力、大中专毕业生、退役军人、城镇新成长劳动力的就业指导和服务。全年新增城镇就业再就业10万人以上，其中失业人员再就业3万人以上。

提高城乡居民收入。激发民众创业致富的激情，支持有条件、有能力的人员投资兴办实业，增加经营性收入。引导民间资本参股、入股各类企业和项目，鼓励群众多形式投资，促进个人资产流动、增值。建立正常的收入增长机制，在发展经济、提高效益的基础上，增加从业人员的工资性收入。落实最低工资保障制度，依法查处违反最低工资标准规定和拖欠职工工资行为。保障农民的土地收益，鼓励农民投资兴办股份合作经济，不断拓宽增收渠道。完善分配机制，调节二次分配，增加中低收入者的收入，扩大中等收入者比重。

完善社会保障体系。进一步扩大社会保险覆盖面，企业养老保险实际缴费职工净增3万人。推进企业年金制度试点，完善医疗保险相关政策规定，制定工伤、生育保险配套政策和农村养老保险经费管理实施办法，推进企业退休人员社会化管理。坚持全面覆盖、分类处理、合理保障，切实解决好失地农民基本生活保障问题。认真落实好最低生活保障措施和五保供养制度，健全社会救助和廉租住房制度，努力保障困难群体的基本生活。完善社区妇女儿童工作网络，切实贯彻老年人优待办法，确保政策措施落实到位。

继续为群众办实事、办好事。今年，要围绕为部分中低收入家庭和困难家庭改善住房、综合整治居民小区环境、增加市区集中供热面积、实施食品药品放心工程等方面，为广大市民办好16件实事。对于这些实事，将集中力量，落实责任，确保年内兑现。

（八）进一步加强精神文明和民主法制建设，构建社会主义和谐社会

提高社会文明程度。大力倡导“爱国守法、明礼诚信、团结友善、勤俭自强、敬业奉献”的基本道德规范，加强社会公德、职业道德和家庭美德教育，积极开展群众性道德实践活动，扩大和完善志愿者服务活动，继续开展爱心捐助活动，发展慈善事业，彰显魅力城市、爱心烟台的城市个性。继续以争创全省全国文明城市为龙头，推进各个领域的精神文明创建活动，全面提高市民素质和文明程度。发挥新闻舆论的导向作用，倡导文明新风，弘扬社会正气。切实搞好国防教育、国防动员、民兵预备役建设和人民防空工作，支持部队现代化建设，更加扎实地开展双拥共建活动，增进军政、军民团结。

营造公平社会环境。坚持科学执政、民主执政和依法执政。建立社情民意反映制度，健全社会公示制度和听证制度，完善专家咨询制度，实行决策论证制和责任制，进一步推进决策的科学化、民主化，加强对权力运行的制约和监督。各级政府要自觉接受人大及其常委会和政协的法律监督、工作监督、民主监督，主动听取各民主党派、工商联、无党派人士及各人民团体的意见建议，认真办好人大代表建议和政协提案。积极推进政务公开、厂务公开和村务公开，拓宽反映社情民意的渠道。坚持从严执政，保证公正执法。规范整顿市场秩序，开展专项检查治理，严厉打击走私贩私和假冒伪劣、价格欺诈、强制消费等不法行为。加强政府信用建设，建立企业、中介机构和个人诚信系统，构建失信处罚机制，着力打造“诚信烟台”。

维护社会安定团结。下大气力抓好“全国最安全城市”建设，着力营造安心、舒心、放心的社会环境。坚决打击境内外敌对势力破坏活动和各类严重刑事犯罪活动，保障国家安全和社会稳定。强化社会治安综合治理，坚持工作重心下移，把“严打”整治斗争、治安防范、矛盾纠纷排查调处、法律服务等工作，落实到城乡基层单位。做好信访工作，积极预防和妥善处置群体性事件，疏导化解人民内部矛盾。全面落实安全生产责任制，实施长效管理，开展专项整治，消除事故隐患，严防重大恶性事故发生。建立健全应急救援和指挥系统，增强对突发事件和各种灾害的处理能力。

三、切实加强政府自身建设

党的十六届四中全会对提高执政能力做出了全面部署，完成新时期的新任务也对提高行政水平提出了更高要求。新的一年，我们必须按照“政治坚定、求真务实、开拓创新、勤政廉洁、团结协调”的要求，不断加强自身建设，进一步增强各级政府的创造力、凝聚力和战斗力。

始终保持奋发有为的精神状态。要认真学习马列主义、毛泽东思想、邓小平理论和“三个代表”重要思想，学习市场经济知识和现代科学文化知识。要深入扎实地开展好保持共产党员先进性教育活动，教育引导各级公务员树立正确的理想信念和高尚的道德情操，增强为民服务的意识，增强干事创业的本领。要善于解放思想，实事求是，永不满足，争创一流，在确立目标、制定决策、推进工作等各个环节，努力体现时代性，把握规律性，富有创造性；善于进行理论思维和战略思维，科学准确地判断形势，及时研究解决改革和发展中的新情况、新问题，做好上级政策与本地实际相结合的文章；善于在各种困难和压力面前，立足自身优势，调动各方面积极性，知难而进，迎难而上，在前进中干事创业；善于应对各种竞争和挑战，及时发现并牢牢把握各种机遇，抢占制高点，争取主动权，努力开创跨越发展的新局面。

大力弘扬求真务实的良好作风。要牢固树立正确的政绩观，牢记“两个务必”，不搞形式主义、官僚主义，不搞弄虚作假、沽名钓誉，不搞任何脱离实际的“形象工程”、“政绩工程”。要严格执行《统计法》和《审计法》，提高统计信息质量，强化审计监督。坚持立党为公、执政为民，切实把群众的呼声和意愿作为指导各项工作的“第一信号”，把关心和服务群众作为各级政府的“第一职责”，把群众的评价作为衡量政府工作的“第一尺度”，千方百计把广大人民的根本利益实现好、维护好、发展好。要大兴调查研究之风，深入基层、深入实际、深入群众，体察实际情况，解决突出问题，切实把以人为本的执政理念体现在各项工作的部署中，落实到经济社会发展的各个方面，努力建设人民满意的政府。

不断完善激励约束的工作机制。要进一步完善决策目标、执行责任、考核监督“三个体系”建设，更好地发挥其在促进工作落实中的规范、引导、激励、约束作用。要坚持实事求是，认真研究和制定科学的奋斗目标，充分调动各方面的积极性、主动性和创造性。要把实现赶超战略的任务目标分解落实到各级各部门，一级抓一级，层层抓落实，切实把各项工作落到实处，抓出成效，干出水平。要本着明确、管用、透明、方便的原则，不断完善各级政府及其部门的政绩评价体系和考核监督办法，为推动政府工作再上新水平提供有力的制度保证。

坚决执行清正廉洁的行为准则。要深入开展理想信念和权力观、地位观、利益观教育，不断提高拒腐防变能力。认真贯彻执行党中央和省、市委关于廉洁自律的各项规定，坚决遏止以权谋私、行贿受贿、为政不廉、行政不公等各种歪风。认真落实廉政承诺，严格执行党风廉政建设责任制。进一步加大行政监察力度，以解决群众反映的突出问题为重点，坚决纠正损害群众利益的不正之风；以查处发生在领导机关和领导干部中滥用权力、谋取私利的违法违纪案件为重点，严厉惩处腐败分子；以杜绝行政过程中腐败行为为重点，进一步完善建设工程、经营性土地出让、产权交易和政府采购等方面的招投标制度，从源头上预防和治理腐败。要加强廉政制度建设，真正形成用制度规范从政行为、按制度办事、靠制度管人的长效机制，保证各级公务员廉洁从政。

各位代表，烟台的发展正面临新的机遇和挑战。要在更高的起点上实现新的跨越，任务更加艰巨，必须付出更大努力。我们要更加紧密地团结在以胡锦涛同志为总书记的党中央周围，高举邓小平理论和“三个代表”重要思想伟大旗帜，在中共烟台市委的坚强领导下，解放思想加压奋进，凝心聚力赶超发展，把烟台改革开放和现代化建设的伟大事业不断推向前进！
